# Plastic Surgery Inclusion in the Undergraduate Medical Curriculum: Perception, Challenges, and Career Choice—A Comparative Study

**DOI:** 10.1155/2017/9458741

**Published:** 2017-05-23

**Authors:** M. Farid, R. Vaughan, S. Thomas

**Affiliations:** ^1^Department of Plastic and Reconstructive Surgery, The Royal London Hospital, Barts Health NHS, Whitechapel Road, London E1 1BB, UK; ^2^Norwich Medical School, University of East Anglia, Chancellor Drive, Norwich NR4 7TJ, UK; ^3^Department of Plastic and Reconstructive Surgery, University Hospital Birmingham NHS Foundation Trust, Queen Elizabeth Hospital Birmingham, Mindelsohn Way, Edgbaston, Birmingham, West Midlands B15 2GW, UK

## Abstract

**Objective:**

The undergraduate medical curriculum has been overcrowded with core learning outcomes with no formal exposure to plastic surgery. The aim of this study was to compare medical students from two educational settings for the basic understanding, preferred learning method, and factors influencing a career choice in plastic surgery.

**Design and Setting:**

A prospective cohort study based on a web-based anonymous questionnaire sent to final year medical students at Birmingham University (United Kingdom), McGill University (Canada), and a control group (non-medical staff). The questions were about plastic surgery: (1) source of information and basic understanding; (2) undergraduate curriculum inclusion and preferred learning methods; (3) factors influencing a career choice. A similar questionnaire was sent to non-medical staff (control group). The data was analysed based on categorical outcomes (Chi-square *χ*2) and level of significance *p* ≤ 0.05.

**Results:**

Questionnaire was analysed for 243 students (Birmingham, *n* = 171/332, 52%) (McGill *n* = 72/132, 54%). Birmingham students (14%) considered the word “plastic” synonymous with “cosmetic” more than McGill students (4%, *p* < 0.025). Teaching was the main source of knowledge for McGill students (39%, *p* < 0.001) while Birmingham students and control group chose the media (70%, *p* < 0.001). McGill students (67%) more than Birmingham (49%, *p* < 0.010) considered curriculum inclusion. The preferred learning method was lectures for McGill students (61%, *p* < 0.01) but an optional module for Birmingham (61%). A similar proportion (18%) from both student groups considered a career in plastic surgery.

**Conclusions:**

Medical students recognised the need for plastic surgery inclusion in the undergraduate curriculum. There was a difference for plastic surgery source of information, operations, and preferred method of learning for students. The study highlighted the urgent need to reform plastic surgery undergraduate teaching in collaboration with national educational bodies worldwide.

## 1. Introduction

There had been an overwhelming expansion in the core knowledge for medical subspecialities. This phenomenon led to the increased competition for plastic surgery inclusion in the undergraduate medical curriculum. The lack of educational opportunities for plastic surgery was noted with the clear decline in curriculum inclusion (13 out of 31 in 1986, 2 out of 34 in 2002) at undergraduate level in the United Kingdom (UK). Such exposure was imperative to help students make informed career decisions at an early stage about career choice [1–3]. Each medical student had unique reasons for pursuing a career in plastic surgery. The interaction with plastic surgeons was deemed the most influential factor while lifestyle and income were less important reasons for medical students [4].

Medical Students were not aware of the basic principles underpinning plastic surgery as a speciality. The complex dimension of “reconstructive” surgery had not been nurtured through medical education. This added to the misconception among public, medical students, and professionals due to the versatility of plastic surgery. Such image was worsened by the media negative role in labelling on plastic surgery as “cosmetic surgery” with rarely a reference to “reconstructive surgery.” Nonetheless, future doctors should match patients' needs through awareness of the skills set offered by plastic surgeons [5]. The concern was public perception which equated plastic surgery with “cosmetic surgery” to be similar to students with no prior undergraduate teaching [6].

The tradition of teaching plastic surgery as an academic subject in the UK started during World War II by Sir Harold Gillies as “a fully fledged and desirable medical school subject for educating undergraduate students” [7]. This historical integration of plastic surgery continued to offer medical students a mixed skills set ranging from the key principles of wound healing to complex clinical scenarios in breast reconstruction, skin cancer, craniofacial anomalies, burns, lower limb trauma, and hand surgery. Nevertheless, neither the preferred learning methods for plastic surgery in the literature nor the reasons for a career choice had been investigated.

The construction of an educational model in plastic surgery was deemed crucial to be aligned with the undergraduate curriculum. The goals were to maximise exposure and help make informed career choices for those interested in pursuing a career in plastic surgery [8].

The main aim of this study was to investigate two different institutions (Birmingham, United Kingdom, and McGill, Canada) for knowledge, learning, and career factors for plastic surgery. It represented a landmark in comparing two unaffiliated universities for medical students underlying perception about plastic surgery.

## 2. Plastic Surgery Undergraduate Curriculum: Birmingham and McGill 

Birmingham University had plastic surgery teaching predominantly incorporated within other surgical specialities. There was no formal teaching in the first two years. During the 4th year, there was an “optional” self-directed four-week clinical placement module. Throughout the 5th year (final year), plastic surgery teaching was in the form of a self-directed learning module without any clinical placement exposure for Birmingham medial students.

McGill University in Canada offered a more formal inclusion of plastic surgery in their curriculum with compulsory lectures and out-patients clinics in plastic surgery during the initial 2 years of medical studies. During 4th and 5th years, students were offered four clinical elective rotations lasting for 4 weeks each in plastic surgery. Overall, McGill students were more likely to be taught plastic surgery during their undergraduate studies compared to Birmingham students. The effect of the increased exposure to plastic surgery was explored in detail as part of this study.

## 3. Methods

### 3.1. Study Design

There was no academic affiliation between the two universities (McGill and Birmingham). The designed survey is based on the identification of integral aspects to plastic surgery teaching and career factors.

The permission to conduct the online survey (13 questions) was gained from both institutions. The questions were related to few parameters about plastic surgery (source of information and generic knowledge, operations, overlap with other specialities, preferred teaching method and clinical attachment period, and encouraging and discouraging factors for a career choice).

The survey was sent via a web-link (Survey Monkey) to final year medical students at Birmingham University (United Kingdom) and McGill University (Canada). There was a “control group” of non-medical administration staff at Queen Elizabeth University Hospital, Birmingham, United Kingdom. The selection was based on considering lay public views without prior medical knowledge or exposure. The exclusion criterion was based on failure to answer the entire survey questions by any student or control group staff.

The null hypothesis assumed no difference in the level of knowledge, method of learning, and career choice among students. All responses for the survey were analysed using SPSS^*∗*^ 15 software (Statistical Package for the Social Sciences). Statistical analysis was performed by a statistician using Chi-square (*χ*2) for categorical data and based on level of significance *p* ≤ 0.05.

### 3.2. Study Aims

The study aimed to evaluate

1. current understanding and source of plastic surgery knowledge;
2. undergraduate curriculum inclusion and the preferred learning method;
3. encouraging and discouraging factors for a career choice.

## 4. Results

**Figure 1:**
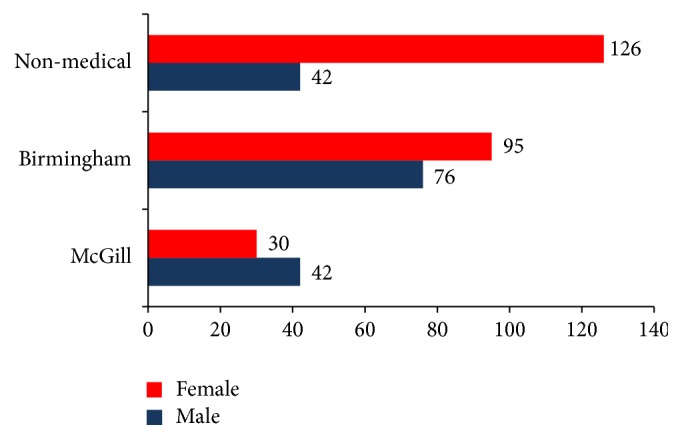
Gender distribution for medical students and non-medical staff.

A total of 243 students completed the questionnaire from both institutions (Birmingham = 171/332, McGill = 72/132), corresponding to 52% and 54%, respectively. The male : female ratio was (M : F 1 : 1.25) in Birmingham compared to (M : F 1.4 : 1) for McGill. The control group (non-medical staff) had 168 respondents (M : F 1 : 3) (Figure 1).

## 5. Birmingham versus McGill Students

### 5.1. Source of Information and Basic Knowledge in Plastic Surgery

Birmingham students mainly acquired their knowledge from the media (70%, *p* < 0.001) while McGill students had more formal teaching (39%, *p* < 0.0001) and exposure to clinical electives (13%, *p* < 0.0001). Friends were more a source of information for McGill (26%) than Birmingham students (16%, *p* < 0.049). The terms “plastic” and “cosmetic” surgery were viewed to be synonymous by only a small number of students at Birmingham (14%) and McGill (4%, *p* < 0.025). Plastic surgery did not have anatomical boundaries for a small number of students (McGill 1%, Birmingham 3%) and both groups were aware of the inclusion for emergency cases (McGill 99%, Birmingham 93%) (Tables 1(a) and 1(b)).

### 5.2. Operation Performed by Plastic Surgeons and Overlap with Surgical Specialities

Operations like facelift, abdominoplasty (tummy tuck), and breast augmentation (breast enlargement) were noticeably related to plastic surgery for both student groups. Skin lesions (McGill 7%, Birmingham 9%) and cleft palate (McGill 26%, Birmingham 21%) were the least linked to plastic surgery. More McGill students considered hand trauma (29%) exclusive to plastic surgeons compared to Birmingham students (12%, *p* < 0.001). A higher proportion of McGill (57%) compared to Birmingham (33%, *p* < 0.001) thought that breast reduction was solely performed by plastic surgeons (Table 1(c)). Although both groups (McGill and Birmingham) acknowledged an interspeciality overlap, it was the least for General surgery (69 & 59%) and most for maxillofacial surgery (93-94%) (Table 1(d)).

### 5.3. Curriculum Inclusion and Preferred Learning Method

McGill students (67%) expressed a stronger desire for plastic surgery inclusion in curriculum compared to Birmingham (49%, *p* < 0.010). The most popular learning methods were lectures (61%) and clinical placement (57%, *p* < 0.001) for McGill students, versus an optional module (61%) for Birmingham students. Both groups rated the optional module similarly (McGill 54%, Birmingham 61%) which ranked 3rd and 1st choice, respectively. Seminars and problem based learning had similar ratios (McGill 24%, Birmingham 26%). A research project was the least popular choice for both groups (McGill 11%, Birmingham 8%). The ideal module duration was 2 weeks (McGill 70%, Birmingham 77%) but the choice of 4 weeks had a lower response rate (McGill 31%, Birmingham 22%) (Tables 2(a) and 2(b)).

### 5.4. Factors Influencing a Career Choice in Plastic Surgery

A similar number of students considered plastic surgery for higher speciality training (McGill 18%, Birmingham 19%). The main encouraging factors for a career choice were specialised skills (McGill 85%, Birmingham 60%, *p* < 0.0001) and clinical diversity (McGill 61%, Birmingham 63%). Patient interaction rated 42% for Birmingham versus 28% for McGill (*p* < 0.04). Research was the least appealing factor for pursuing a plastic surgery career (McGill 15%, Birmingham 11%). Nearly two-thirds of both groups shared the lack of interest as a reason and over half the students were discouraged by the long training period and working hours. Working with other surgical specialists did not influence a career choice in plastic surgery (Tables 3(a) and 3(b)).

### 5.5. McGill and Birmingham Students Group versus Control Group

#### 5.5.1. Source of Information and Basic Knowledge in Plastic Surgery

The main source of information was the media for the control group (85%, *p* < 0.0001) and Birmingham (70%) compared to McGill (31%). Friends were another common source of information for McGill (26%), Birmingham (16%), and control group (15%, *p* < 0.0001). Teaching and electives rated higher for McGill students compared to Birmingham (*p* < 0.0001) (Table 1(a)).

The control group perceived the term “cosmetic” to be synonymous with “plastic” (31%, *p* < 0.001) more than students (McGill 4%, Birmingham 14%). All 3 groups considered plastic surgery to have no anatomical boundaries (Control 7%, McGill 1%, and Birmingham 3%). Students (McGill 99%, Birmingham 93%) thought that plastic surgery involved emergency cases but a lower response for the control group (80%, *p* < 0.0001) (Table 1(b)).

The control group considered that 75% of medical students should apply for plastic surgery training while students had a significantly lower apply rate (McGill 18%, Birmingham 19%, *p* < 0.001) (Table 1(b)).

### 5.6. Operations Performed by Plastic Surgeons and Overlap with Surgical Specialities

Medical students (McGill 57%, Birmingham 61%) responses were higher than the control (46%, *p* < 0.008) for tummy tuck (abdominoplasty). A similar pattern was observed for facelift, cleft palate, hand trauma, skin lesions, and breast enlargement (*p* < 0.01). There was no significant difference (*p* > 0.05) for the 3 groups response to breast reduction and prominent ear procedures (Table 1(c)).

The students had a higher response for all subspeciality overlap compared to the control group (*p* < 0.0001). Trauma and orthopaedics, maxillofacial surgery, ENT, and dermatology were the top four overlapping surgical subspecialties choices for McGill and Birmingham students but had a consistently lower rating among control group (*p* < 0.001) (Table 1(d)).

All 3 groups preferred lectures, clinical placement, and an optional module as the top three learning methods. Lectures rated 61%, 38%, and 50% for McGill, Birmingham, and control group, respectively. Clinical placement (64%) was the 1st choice for control group (*p* < 0.001), 2nd for McGill (57%), and 3rd for Birmingham (37%, *p* < 0.0001). A research project was the least favourable choice in all three groups, with a higher response for control group (27%, *p* < 0.0001) versus students (McGill 11%, Birmingham 8%) (Table 2(a)).

The control group (71%, *p* < 0.001) and students (McGill 67%, Birmingham 49%) shared the view that plastic surgery should be taught in the undergraduate curriculum. A clinical rotation for 5-6 weeks was the appropriate learning duration for control group (62%, *p* < 0.0001) but students opted for 2-week placement (McGill 69%, Birmingham 77%) (Table 2(b)).

### 5.7. Factors Influencing a Career Choice in Plastic Surgery

The most encouraging factor for a career in plastic surgery was specialised skills for the control group (78%, *p* < 0.01) and McGill students (85%). Clinical diversity was more popular for students (McGill 61%, Birmingham 63%) than the control group (35%, *p* < 0.001). Patient interaction was similar at 42% for both the control group and Birmingham compared to 28% for McGill (*p* < 0.0001). Research was the least encouraging factor among students (McGill 15%, Birmingham 12%) and control (23%) as a career incentive (Table 3(a)).

The discouraging factors were long working hours (32%) and long training (16%) for the control group (*p* < 0.001) with a higher response in Birmingham (52%, 60%) and McGill (46%, 54%), respectively. Working with other specialists was the least discouraging factor for the control group, McGill and Birmingham (4%, 1%, and 2%, resp.). Two-thirds of all 3 groups considered the lack of interest in plastic surgery as the most discouraging factor (Table 3(b)).

## 6. Discussion

The main aim of this study was to determine the perception of medical students of plastic surgery, undergraduate curriculum inclusion, and influencing career choice. Students' prior clinical exposure, knowledge, and educational needs formed the basis for responses.

### 6.1. Source of Information and Basic Knowledge in Plastic Surgery

The name plastic originated from the Greek* “plastikos”* or Latin* “plasticus,”* meaning to mould. Nevertheless, the meaning of the word had been misunderstood by medical students. More Birmingham students than McGill considered the term “plastic” and “cosmetic” to have a similar meaning. The concerning fact that the knowledge about plastic surgery for students was predominantly from the media shared similar views to non-medical staff. This was echoed by Hamilton III et al. (2004) who revealed that medical students gained plastic surgery knowledge in equal proportion from the media and formal medical school teaching. On the other hand, McGill students had formal teaching and clinical electives throughout undergraduate years and a third of their intake did a plastic surgery rotation in their final year. This explained the significantly lower response for media as a source of information for this group [9–11].

Cosmetic surgery was perceived to be technically less challenging, associated with less operative risk, a shorter recovery, and less postoperative pain compared to “plastic or reconstructive” surgery. Reid and Malone (2008) reiterated that the media had a tendency to misrepresent cosmetic surgery as plastic or reconstructive surgery because the term “cosmetic” was considered synonymous to “plastic” or “reconstructive” surgery [12].

The funding of cosmetic surgery by the national health system was considered acceptable by non-medical staff more than Birmingham or McGill students. This fact reflected on the different understanding for the meaning of cosmetic surgery. The issue of funding remains a matter of political contention due to the financial challenges to meet patients' clinical needs [13, 14].

### 6.2. Operations Performed by Plastic Surgeons and Overlap with Surgical Specialities

The scope of surgical operations performed solely by plastic surgeons had not been determined in-depth in terms of students' perception. A relevant study by Agarwal et al. (2014) reported the poor understanding of medical students about the role of plastic surgeons for hand surgery. As part of the study, a list of operations was included to whether performed solely by plastic surgeons. Face lifts, tummy tucks, and breast enhancement were recorded as the top three operations for all three groups while skin lesions, hand trauma, and cleft surgery were the bottom three operations. There had been no limit to the range of plastic surgery operations and the extent of pathological breadth from the crown of the head to the sole of the foot. Hence, the expected overlap with different surgical subspecialties was highlighted in the 3 groups response. Maxillofacial surgery ranked the highest for Birmingham and control group versus ENT for McGill. Vascular surgery ranked the least for the control. Medical students were more aware of emergency cases in plastic surgery than the control group. This trend supported Agarwal et al. (2013) who concluded the need to improve medical students' education about the scope of plastic surgery [9, 15–17]. It seemed apparent that students showed better awareness of the overlap with other specialities than control group. Another pattern was noted for the type of operations performed by plastic surgeons. Breast enhancement was one of the top choices for all groups while breast reduction ranked lower for Birmingham students and control group compared to McGill. This difference highlighted the in-depth differing perception between students groups due to the underlying level of teaching and exposure to plastic surgery.

The scope of undergraduate teaching, curriculum inclusion, and preferred method of plastic surgery teaching was supported by students and control group. Nevertheless, there was a variable difference for these parameters among students and control group. Seminars were more valued among the control than students. A research project was the least popular teaching method for students and ranked last for control group. Lectures and clinical placement were more popular among McGill and noncontrol group compared to Birmingham. All these modalities in terms of didactic lectures, electives, and clinical rotations can be supported with career workshops, open days, and e-learning modules [10]. Nonetheless, this should be tailored and explored with students during early undergraduate years prior to deciding the range of teaching methods. The teaching could be targeted through subspeciality areas like burns, head and neck, skin and breast cancer, trauma, congenital anomalies, and their reconstruction. These are often linked to many “charities” that medical students can join earlier in their medical journey for long term mutual benefit.

An optional module was one of the top choices for all 3 groups but the interesting aspect was that the preferred duration was 2 weeks for students compared to 6 weeks for the control. It reflected on students understanding of the challenges in curriculum to accommodate a longer rotation and the lack of sufficient hospital allocations for continually increased student intake [18, 19]. Regardless of the clinical rotation period, the exposure to a plastic surgery ward would give medical students the opportunity to observe a range of pathologies including lumps, ulcers, pressure sores, lymphadenopathy, head and neck masses, burns, and soft tissue injuries. This knowledge is deemed essential for today's doctors to appreciate the scope of plastic surgery.

### 6.3. Factors Influencing a Career Choice

The students showed a similar response for a career in plastic surgery. One of the potential factors would be a varied possible personal preference to other specialities. This was an unusual finding considering that McGill students have more exposure and teaching for the speciality. Mahalingam et al. (2014) reported that increased plastic surgery exposure during undergraduate studies correlated with a career choice [20]. We further investigated the influencing factors to choose plastic surgery as a career. Specialised skills seemed a commonly favourable encouraging factor for all 3 groups. Research was the least popular reason for a career choice. This theme should be considered by educators to meet students' needs as it also meets public opinion. Similarly, a lack of interest was most discouraging factor for a career choice in all 3 groups. Long training and lengthy working hours were less appealing for students than the control group. Previous work by Ek et al. (2005) reiterated that the increasing desire of medical students to maintain a healthy work-life balance had reduced the choice of surgery as a career. May et al., 2005, also reported that this was associated with a better sense of career satisfaction. The perception of a better lifestyle associated with cosmetic private practice was a motivating factor among student in previous research. Working with other specialties did not discourage students or the public reflecting the awareness for team work in present day medicine [21–23]. All groups were unified in research being the most discouraging reason for a career in plastic surgery. The latter fact was striking as research had been deemed key to a surgical career choice and progression. Students preferred to pursue a career without undertaking research who put more value on clinical diversity and skills.

The phenomenon regarding why students made such choices can be underlined by exploring their medical curriculum. McGill University encouraged student participation in plastic surgery in all academic years which was endorsed by the Canadian postgraduate training scheme. Students at McGill were better informed through enhanced teaching and clinical placements to make a career choice earlier. On the other hand, the structure of medical training in the UK might influence Birmingham students' choice. Wade et al. (2009) reported that most plastic surgeons (67%) in the United Kingdom chose their career during postgraduate training. It strengthens the rationale that exposure to plastic surgery further helps such choice which is likely to be in postgraduate period in the UK. Other researchers like Sutton et al. (2014) reported a recent shift in the United Kingdom that a similar percentage of medical students (65%) were choosing their specialty earlier at undergraduate medical studies. Hence, the lack of exposure to plastic surgery at the undergraduate level was deemed a disadvantage for those interested in such choice [24–26].

In terms of gender variation, surgical specialities may not be popular among female students possibly due to lack of interest and work-life balance. Few strategies can be implemented to encourage higher enrolment among this group. Role models, mentoring, a positive clinical posting, and early exposure to career fairs and taster days positively influenced female trainees to choose a surgical career. There is a current trend over recent years that more female trainees are encouraged to choose surgery as a career in the future. This has been endorsed by the Royal Colleges and national higher surgical training scheme [27–30].

The strengths of this project were multifaceted and aimed to raise global awareness about plastic surgery teaching and career choice. There has been no previous research comparing two educational settings for their underlying knowledge in relation to curriculum exposure. Previous literature highlighted the issue for the lack of teaching in plastic surgery but failed to determine a causal link to curriculum or career factors. The two institutions were not affiliated in any academic setting so there is no bias in this research. The study was validated by comparing the student group to non-medical staff to strengthen the finding for curriculum comparison and career choice. It highlighted the need for a more formal introduction of plastic surgery teaching at an early stage for medical students. Nevertheless, few weaknesses were inherent to the nature of any survey based cohort study. The response rate was over 50% for students at both institutions and deemed acceptable. The authors did not think that a higher response would achieve considerably a variable outcome but may strengthen the study findings. This statement is supported by the clear statistical difference for obtained results. Another weakness was the study represented a snapshot for final year students at two institutions. Further research should be considered worldwide for a greater in-depth understanding for the variable patterns in teaching and career choice. This would be truly important for countries with a considerable practice in plastic surgery and more public awareness (e.g., Brazil, Korea, and USA) [31, 32]. Furthermore, our study determined the public opinion for the United Kingdom only. It would be more valuable to collect data from Canadian public even though the aim was not to compare public views.

The study also determined the need for medical schools, surgeons, and students to work in close partnership to deliver the best plastic surgery education. A good example of such partnership was “The Medical Student Learning Centre,” a web-based project aimed at teaching plastic surgery delivered by the Dalhousie Faculty of Medicine, Division of Plastic and Reconstructive Surgery, Halifax, Canada [33]. This method ensured a diverse learning experience within the limited time in the undergraduate curriculum and set an example for future learning. The emphasis for this platform was on providing both formal and self-directed undergraduate teaching in plastic surgery. Another suggestion from this study would be to integrate plastic surgery with other curriculum components. This crucial step was initiated by the Canadian Carnegie Foundation (2004) with recommendations for the advancement of teaching in undergraduate medical education. It emphasised the need to standardise learning outcomes, integrate formal knowledge with clinical experience, and focus on progression of professional identity [34–39].

## 7. Conclusions

This study was a snapshot of medical students' perception and teaching of plastic surgery in the United Kingdom and Canada. Students at both medical schools had a different level of understanding about plastic surgery and factors for career choice which was reflected by their undergraduate medical curriculum.

Educational and regulatory organisations exemplified by the Royal College of Surgeons in both countries, the British Association of Plastic & Reconstructive Surgeons (BAPRAS), and the Canadian Society of Plastic Surgeons need to introduce and monitor formal plastic surgery teaching at the undergraduate level within the curriculum. These organisations should liaise with key educators including the Deans of medical schools and government institutions to facilitate this process.

## Figures and Tables

**Table 1a:** (a) Source of information for plastic surgery

Options	McGill % (*n*)	Birmingham % (*n*)	*χ2*, * p value *	Non-medical staff % (*n*)	*χ2*, *p value *
Elective	12.5 (9)	2.3 (4)	*10.50*, *p* < 0.001	7.74 (13)	NS
Media	30.55 (22)	69.59 (119)	*30.41*, *p* < 0.001	84.52 (142)	*32.25, p* < 0.0001
Teaching	38.89 (28)	17.54 (30)	* 13.06*, *p* < 0.0001	3.57 (6)	31.12, *p* < 0.0001
Friends	26.39 (19)	15.79 (27)	3.87, *p* < 0.049	14.88 (25)	* 29.95, p* < 0.0001

**Table 1b:** (b) Generic knowledge of plastic surgery

Question	McGill % (*n*)	Birmingham % (*n*)	*χ*2, *p* value	Non-medical staff % (*n*)	*χ*2, *p* value
Is the name “plastic” the same as “cosmetic”?	4.17 (3)	14.04 (24)	*4.99, p* < 0.025	30.95 (52)	* 25.18*, *p* < 0.0001
Is plastic surgery limited to a specific part of the body?	1.4 (1)	2.92 (5)	NS	6.55 (11)	NS
Do plastic surgeons deal with emergency cases?	98.61 (71)	92.98 (159)	NS	79.76 (134)	* 21.739*, *p* < 0.0001
Should plastic surgery be included in the undergraduate curriculum?	66.67 (48)	48.54 (83)	*6.70, p* < 0.010	70.83 (119)	* 11.94*, *p* < 0.001
Is it appropriate to offer cosmetic surgery operations under the National Health Service?	40.27 (29)	56.14 (96)	*5.10, p* < 0.024	69.05 (118)	* 14.70*, *p* < 0.001
Would you/students consider applying for plastic surgery training?	18.06 (13)	18.71 (32)	NS	75.00 (126)	*130.43*, *p* < 0.0001

**Table 1c:** (c) Operations performed by plastic surgeons only

Options	McGill % (*n*)	Birmingham % (*n*)	*χ2*, *p value*	Non-medical staff % (*n*)	*χ2*, *p value*
Prominent ear	37.5 (27)	43.86 (75)	NS	43.86 (73)	NS
Tummy tuck	56.94 (41)	60.82 (104)	NS	46.43 (78)	*7.02*, *p* < 0.008
Cleft palate	26.39 (19)	21.05 (36)	NS	38.69 (65)	* 12.39*, *p* < 0.0001
Hand trauma	29.17 (21)	12.28 (21)	*10.10*, *p* < 0.001	28.57 (48)	* 7.4*, *p* < 0.007
Facelift	72.22 (52)	76.02 (130)	NS	60.71 (102)	* 9.36*, *p* < 0.002
Skin lesions	6.94 (5)	8.77(15)	NS	28.57 (48)	* 29.76*, *p* < 0.0001
Breast Enlargement	70.83 (51)	60.23 (103)	NS	51.79 (87)	* 5.5*, *p* < 0.019
Breast reduction	56.94 (41)	33.33 (57)	*11.74*, *p* < 0.001	41.66 (70)	NS

**Table 1d:** (d) Surgical specialties overlap with plastic surgery

Options	McGill % (*n*)	Birmingham % (*n*)	*χ2*, *p value *	Non-medical staff % (*n*)	*χ2*, *p value *
Trauma and orthopaedics	88.89 (64)	87.13 (149)	NS	69.64 (117)	* 20.36*, *p* < 0.0001
Vascular	69.44 (50)	62.57 (107)	NS	23.21(39)	* 68.23*, *p* < 0.0001
General surgery	69.44 (50)	59.65 (102)	NS	43.06 (72)	* 15.53*, *p* < 0.0001
Dermatology	88.89 (64)	88.3 (151)	NS	55.36 (93)	* 58.02*, *p* < 0.0001
Ear, nose, throat (ENT)	93.06 (67)	80.7 (138)	*5.86*, *p* < 0.015	56.55 (95)	* 38.98*, *p* < 0.0001
Maxillofacial	91.67 (66)	94.74 (162)	NS	79.17 (133)	* 19.97*, *p* < 0.0001

**Table 2a:** (a) Method for plastic surgery teaching

Options	McGill % (*n*)	Birmingham % (*n*)	*χ*2, *p* value	Non-medical staff % (*n*)	*χ*2, *p* value
Seminars	23.61 (17)	25.73 (44)	NS	40.48 (68)	*10.91*, *p* < 0.001
Clinical attachment	56.94 (41)	36.84 (63)	*8.363*, *p* < 0.004	64.29 (108)	*18.36*, *p* < 0.0001
Lectures	61.11 (44)	38.01 (65)	*10.93*, *p* < 0.001	50 (84)	NS
Problem based learning	15.72 (11)	13.45 (23)	NS	36.9 (62)	*29.13*, *p* < 0.0001
Research project	11.11 (8)	8.12 (14)	NS	27.38 (46)	*42.41*, *p* < 0.0001
Optional module	54.17 (39)	61.4 (105)	NS	42.86 (72)	NS

**Table 2b:** (b) Duration for an optional module in plastic surgery

Weeks	McGill % (*n*)	Birmingham % (*n*)	*χ*2, *p* value	Non-medical staff % (*n*)	*χ*2, *p* value
0–2	69.44 (50)	76.74 (132)	NS	7.14 (12)	*182.96*, *p* < 0.0001
3-4	30.56 (22)	21.64 (37)	NS	30.36 (51)	NS
5-6	0%	1.17 (2)	NS	61.9 (104)	*193.63*, *p* < 0.0001

**Table 3a:** (a) Encouraging factors for a career in plastic surgery

Options	McGill % (*n*)	Birmingham % (*n*)	*χ*2, *p* value	Non-medical staff % (*n*)	*χ*2, *p* value
Patient interaction	27.78 (20)	42.11 (72)	*4.42*, *p* < 0.036	42.26 (71)	NS
Clinical diversity	61.11 (44)	63.16 (108)	NS	34.52 (58)	*31.23*, *p* < 0.0001
Research	15.28 (11)	11.69 (20)	NS	22.62 (38)	NS
Specialised skills	84.72 (61)	59.64 (102)	*14.42*, *p* < 0.0001	77.98 (131)	*5.79*, *p* < 0.016

**Table 3b:** (b) Discouraging factors for a career in plastic surgery

Options	McGill % (*n*)	Birmingham % (*n*)	*χ*2, *p* value	Non-medical staff % (*n*)	*χ*2, *p* value
Long working hours	45.83 (33)	52.05 (89)	NS	32.14 (54)	*13.27*, *p* < 0.0001
Lack of interest	68.06 (49)	64.32 (110)	NS	67.26 (113)	NS
Long training	54.17 (39)	59.65 (102)	NS	38.1 (64)	*15.78*, *p* < 0.0001
Working with other specialists	1.40 (1)	2.34 (4)	NS	4.17 (7)	NS

*Note*. NS = not significant, *p* > 0.05.
